# Association between Gut Microbiota and Muscle Strength in Japanese General Population of the Iwaki Health Promotion Project

**DOI:** 10.3390/microorganisms12030622

**Published:** 2024-03-20

**Authors:** Yoshikuni Sugimura, Yichi Yang, Akira Kanda, Akihiro Mawatari, Yoshinori Tamada, Tatsuya Mikami, Shigeyuki Nakaji, Kazushige Ihara

**Affiliations:** 1 Department of Social Medicine, Graduate School of Medicine, Hirosaki University, 5 Zaifu-cho, Hirosaki 036-8562, Aomori, Japan; y.sugimura@hirosaki-u.ac.jp (Y.S.); yangy@hirosaki-u.ac.jp (Y.Y.); sips-museum0128@hotmail.co.jp (A.M.); nakaji@hirosaki-u.ac.jp (S.N.); 2 Department of Nutrition, Faculty of Health Sciences, Graduate School of Health Sciences, Aomori University of Health and Welfare, 58-1 Mase, Hamadate 030-8505, Aomori, Japan; a_kanda@auhw.ac.jp; 3 Department of Medical Data Intelligence, Hirosaki University, 5 Zaifu-cho, Hirosaki 036-8562, Aomori, Japan; y.tamada@hirosaki-u.ac.jp; 4 Department of Preemptive Medicine, Innovation Center for Health Promotion, Graduate School of Medicine, Hirosaki University, 5 Zaifu-cho, Hirosaki 036-8562, Aomori, Japan; tmika@hirosaki-u.ac.jp

**Keywords:** gut microbiota, muscle strength, grip strength/BMI, sarcopenia

## Abstract

The association between the gut microbiota and muscle strength has garnered attention in the context of mitigating muscle decline. However, many study subjects have been individuals with existing illnesses or the elderly only. This study aims to elucidate the association between the gut microbiota and muscle strength indicators using grip strength/BMI in a large-scale study of community residents. The mean age of men (*n* = 442) and women (*n* = 588) was 50.5 (15.3) and 51.2 (15.9) years, respectively. The muscle strength indicator used was grip/BMI. The association between total read count and genus-level gut microbiota and muscle strength was analyzed. The mean grip/BMI was 1.8 (0.3) for men and 1.2 (0.2) for women. The genus of the gut microbiota that showed an association in both sexes was *Eggerthella* (men: *β* = 0.18, CI: 0.04–0.31, *p* = 0.009; women: *β* = 0.07, CI: 0.00–0.12, *p* = 0.028). *Blautia*, *Eggerthella* and *Faecalibacterium* were found to be significantly associated with grip/BMI in both the multiple regression analysis and Spearman’s correlation analysis after the multiple comparison adjustment. These results suggest that an increase in *Blautia* and *Eggerthella*, coupled with a decrease in *Faecalibacterium*, may contribute to muscle strengthening or the suppression of muscle weakness.

## 1. Introduction

The gut microbiota is involved in the regulation of other biological functions such as immune function, enzyme activity, and hormone secretion [1]. The gut microbiota forms a complex ecosystem, and the composition, profile, and diversity of the gut microbiota as an aggregate reflect the health status of the host [2]. The loss of intestinal microbiota diversity and the associated imbalances can cause immune allergies and metabolic disorders, leading to obesity, metabolic syndrome, and lifestyle-related diseases [3,4]. On the other hand, the gut microbiota also plays a role in the adaptation of compositional profiles due to aging, dietary habits, or drug metabolism [5].

It has been suggested that physical activity alters the composition and function of the intestinal microbiota [6,7,8,9,10]. While physical activity is related to muscle strength, recently, some studies have reported that particular types of gut microbiota are associated with muscle strength. Fielding et al., focusing on the elderly, indicated that the high lower-limb strength group had a higher relative abundance of *Prevotella* and *Barnesiella* compared to the low lower-limb strength group [11]. Another study has shown a positive correlation between *Lachnospira*, *Eubacterium*, *Ruminococcus*, and grip strength [12]. Muscle strength is related to morbidity and mortality [13,14]. Therefore, the enhancement of muscle strength with exercise or other physical activities has been a significant challenge for public health. This is particularly relevant for the aging population and individuals with disabilities, who may have restricted physical capabilities. A specific genus of the gut microbiota that is discovered to be related to muscle strength might be a potential target for the development of probiotic or prebiotic interventions, thereby contributing to the broader discourse on maintaining muscle strength. Elucidating this discovery might offer valuable insights for the mechanisms underlying the relationship between the gut microbiota and muscle strength. Although the two previous studies seem to have great potential for expansion, they are small sample studies and do not sufficiently consider confounding factors; exercise is an important confounder as exercise is associated with muscle strength and may be associated with the gut microbiota.

Grip strength, which is commonly used to measure muscle strength, has been found to have a positive correlation with overall muscle strength and lower-limb strength [15,16,17], as well as a positive correlation with height and weight [18]. Therefore, there is a possibility that the muscle strength evaluation of taller individuals or those who are overweight may appear higher. Chun’s report indicated a negative association between grip strength/BMI and the prevalence of metabolic syndrome and a positive association with a higher quality of life [19]. Furthermore, a lower grip strength/BMI was associated with an increased risk of sarcopenia and a decrease in walking speed [20]. Muscle strength evaluated using grip strength/BMI is suitable for large-scale studies targeting a wide range of age groups.

We have conducted a large-scale survey of the local population, allowing for comprehensive consideration of health-related indicators, including dietary intake, physical function measures, and exercise habits. The aim of this study is to elucidate the association between the gut microbiota and muscle strength indicators using grip strength/BMI, considering confounding effects of exercise habits and health-related indicators, in using the data from the survey.

## 2. Materials and Methods

### 2.1. Study Participants

This study was a cross-sectional study. The study consisted of 1475 participants (1073 in 2017, 244 in 2018, and 158 in 2019 after duplicate elimination) of the Iwaki Health Promotion Project health check-up [21]. For duplicate participants, measurement records from the first year were adopted. Participants in the following categories were also excluded: (1) lack of height and body weight measures (*n* = 10), (2) lack of both left and right grip strength measures (*n* = 385), (3) lack of gut microbiota measures (*n* = 38), (4) lack of complete Brief Diet History Questionnaire (BDHQ) (*n* = 2), and (5) incomplete self-administered questionnaire (*n* = 10). The final number of study participants amounted to 1030 (men: 442; women: 588).

### 2.2. Measurements of Muscle Strength

Muscle strength in this study was measured using a Smedley-style analog handgrip dynamometer (T.K.K.5001, Takei Scientific Instruments Co., Ltd., Niigata, Japan). Grip strength measurements were taken with the participants in an upright position, with their arms naturally hanging down, and were instructed to grip the dynamometer. Participants were requested not to perform any actions such as “pressing against the body” or “swinging”. The grip width of the dynamometer was adjusted so that the second joint of the index finger was at approximately 90 degrees. Grip strength measurements were conducted twice, alternately for each hand. The recorded values were rounded down to the nearest kilogram, and the average of the better of the left- and right-side records was used, respectively. The intraclass correlation for grip strength in this study was highly reproducible: right 0.926 (CI: 0.917–0.935) and left 0.923 (CI: 0.914–0.932). Previous studies reported intraclass correlations of grip strength measurements ranging from 0.90 to 0.97, indicating the utility of the measurement method used in this study [22,23].

### 2.3. Measurements of Body Composition

The body composition assessment in this study was conducted using measurements of height and weight. Height (cm) and body weight (kg) were measured to the first decimal place. Body weight is somewhat affected by changes in body water percentage and activities such as eating, sweating, and urinating during the day. Therefore, participants were asked to skip breakfast on the day of body composition measurements to minimize diurnal behavioral variation. To minimize the variabilities of the measuring instrument, the same instrument was used throughout the study (MC-190, Tanita, Tokyo, Japan). The participants’ body mass index (BMI) (kg/m^2^) was calculated by dividing the weight (kg) by the square of the height (m).

### 2.4. Measurements of the Gut Microbiota

Fecal sampling kits were distributed to the participants before the Iwaki Health Promotion Project health check-up. The fecal sampling kit contained guanidine thiocyanate solution (100 mM Tris-HCl (pH 8.0), 40 mM Tris-EDTA (pH 8.0), 4 M guanidine thiocyanate, and 0.001% bromothymol blue) (TechnoSuruga Lab Co., Ltd., Shizuoka, Japan) for the stability of the gut microbiota composition. Participants were instructed to collect the fecal samples at their homes using the kit from three days before the health check-up to the day of the check-up. The collection method instructed defecating onto toilet paper and using the provided spoon to transfer the feces into the container. Participants were instructed to store the samples in their home refrigerators until the day of the health check-up. The fecal samples were stored at 4 °C for three months at TechnoSuruga Lab until DNA extraction [24,25,26].

The DNA of the gut microbiota was extracted using the zirconium bead-beating method. Automated nucleic acid extraction equipment (Precision System Science, Chiba, Japan) and MagDEA DNA200 (Precision System Science) were used for DNA purification. Amplification of DNA was adjusted to a concentration of 10 ng/μL using the NanoDrop spectrophotometric method.

The V3-V4 region of the prokaryotic 16S rDNA of the gut microbiota was amplified using the Universal Primer set [27]. The amplified DNA sequences were determined using the Illumina MiSeq sequencing system and MiSeq Reagent Kit v3 (Illumina, San Diego, CA, USA).

Partial base sequences of the 16S rDNA (approximately 380–430 bp) were clustered with a similarity rate of over 97% using VSEARCH (version 2.4.3). Clusters identified with a confidence level of less than 0.8 were predicted and grouped as unclassified. The classification of clusters was estimated using the standard classification predicted by the RDP classification method. The number of each taxonomic group was calculated as the lead counts of the partial base sequences of 16S rDNA. The relative abundance (%) of each genus in the gut microbiota was calculated by dividing the read count of each genus by the total count. No temporal trends were observed for individual variations, including seasonal and daily fluctuations [28].

### 2.5. Self-Reported Questionnaires

For the dietary survey, the self-administered Brief Diet History Questionnaire (BDHQ) was used to calculate the daily intake of protein, fat, carbohydrates, total dietary fiber, and alcohol [29]. Additionally, information on lifestyle habits was obtained from the self-administered questionnaires, including responses on habitual medicine use (Yes/No), smoking (Yes/No), exercise (Yes/No), and sleep time (min/day).

### 2.6. Statistical Analysis

All analyses were stratified by gender. The characteristics of the participants were presented as mean ± standard deviation for continuous variables and as frequencies and percentages for nominal variables.

The analysis of the gut microbiota involved conducting the Mann–Whitney U-test on the absolute total read count data. In addition, at the genus level, taxa with a relative abundance greater than 0.1% were included in the analysis. Finally, 54 genera were included in the analysis from a total of 413 genera. The Mann–Whitney U-test was used to examine gender differences.

The association between gut microbial genus (outcome) and grip/BMI (exposure) was investigated by a multivariate analysis using a linear regression model. This multivariate analysis was adjusted for age, nutritional intake (protein, fat, carbohydrates, total dietary fiber, and alcohol), habitual medicine use, smoking, exercise, and sleep time.

The correlation between gut microbial genus and grip/BMI was examined using Spearman’s correlation. In addition, to determine the gut microbial genus associated with grip/BMI, a false discovery rate (FDR) correction was performed. The FDR was controlled using the Benjamini–Hochberg procedure to adjust for multiple testing, and significance was set at <0.05 [30].

Statistical analysis was conducted using SPSS (version 25; SPSS Inc., Chicago, IL, USA), and statistical significance was set at *p* < 0.05.

## 3. Results

### 3.1. Participant Characteristics

The characteristics of the participants are shown in Table 1. The mean age of men (*n* = 442) was 50.5 ± 15.3 years, and the mean age of women (*n* = 588) was 51.2 ± 15.9 years. Approximately half of both men and women were taking prescribed medication from physicians. In addition, less than 30% of participants reported having an exercise habit.

### 3.2. Characteristics of the Gut Microbiota

The total read count of the participants’ gut microbiota and the relative counts of 54 genera are shown in Table 2. The mean total read count was 20,395.0 ± 5244.9 for men and 20,505.4 ± 55,536.7 for women, and there was no significant difference between men and women (*p* < 0.989). Among the 54 genera, significant differences in the mean relative counts were observed between men and women for 25 genera. The top three genera with the highest mean relative counts (*Bacteroides*, *Bifidobacterium*, and *Blautia*) were higher in women than in men, but the differences were not significant. In addition, among the top 10 genera with the highest mean relative counts, *Prevotella* (*p* < 0.001) and *Collinsella* (*p* < 0.001) were significantly higher in men compared to women. On the other hand, women had significantly higher levels of *Faecalibacterium* (*p* < 0.001) and *Ruminococcus* (*p* < 0.001) compared to men. *Ruminococcus2*, *Anaerostipes*, and *Roseburia* were higher in men than in women, but the differences were not significant.

### 3.3. Association between Gut Microbiota and Muscle Strength

The results of the multiple linear regression analysis (with forced entry of independent variables) with grip/BMI as the dependent variable are shown in Table 3. The analysis included all 10 adjustment variables, including age, nutritional intake (protein, fat, carbohydrates, total dietary fiber, and alcohol), habitual medicine use, smoking, exercise, and sleep time. Ten genera were shown to have an effect on muscle strength in men. Five of the genera showed positive effects (*Blautia: β* = 0.01, CI: 0.00–0.02, *p* = 0.021; *Clostridium XVIII: β* = 0.03, CI: 0.00–0.05, *p* = 0.038; *Eggerthella: β* = 0.18, CI: 0.04–0.31, *p* = 0.009; *Erysipelotrichaceae_incertae_sedis*: *β* = 0.03, *CI*: 0.00–0.05, *p* = 0.027; *Escherichia Shigella: β* = 0.02, CI: 0.00–0.04, *p* = 0.041). The effect of three bacterial genera was shown in women. Two of the genera showed positive effects (*Eggerthella*: *β* = 0.07, CI: 0.01–0.12, *p* = 0.028; *Ruminococcus*: *β* = 0.00, CI: 0.00–0.01, *p* = 0.047). The genus of the gut microbiota that showed an association in both men and women was *Eggerthella*.

**Figure 1:**
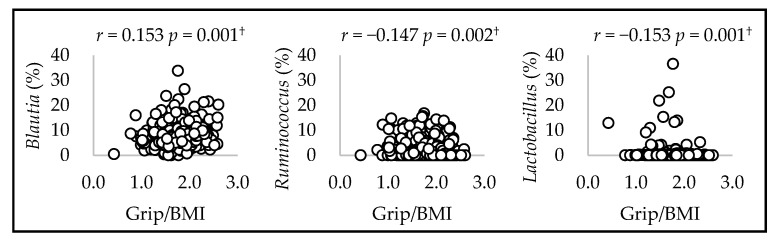
Spearman’s correlation between mean relative counts and grip/BMI of the three genera in men. ^†^ *p*-values that passed the FDR 0.05 threshold.

**Figure 2:**
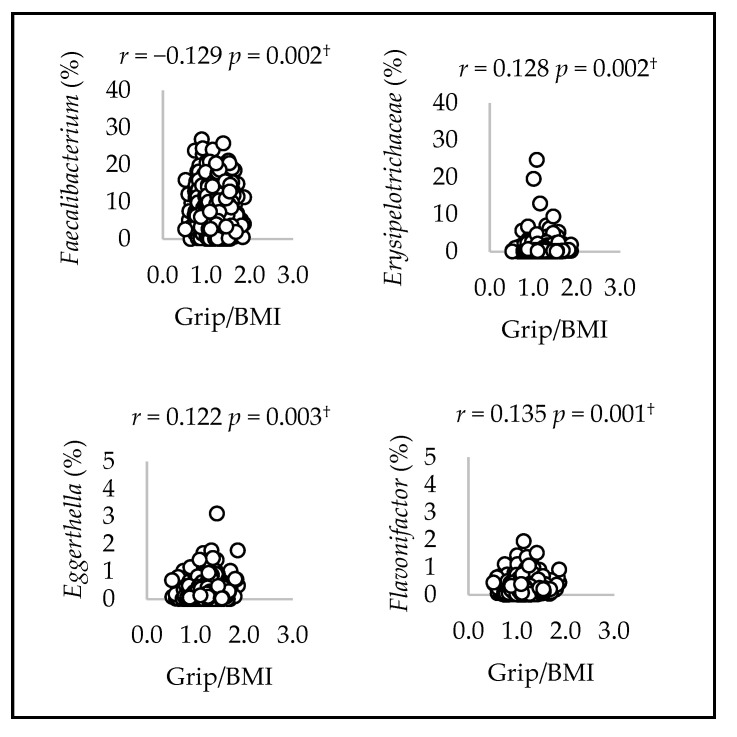
Spearman’s correlation between mean relative counts and grip/BMI of the fourth genera in women. ^†^ *p*-values that passed the FDR 0.05 threshold.

Scatter plots illustrating the significant correlations between gut microbial genera and grip/BMI with FDR < 0.05 are shown in Figure 1 and Figure 2. The correlations between gut microbial genera and grip/BMI were observed for 3 out of 54 genera in men and 4 out of 54 genera in women.

Grip/BMI was positively correlated with *Blautia* in men (*r* = 0.153, *p* = 0.001). In addition, grip/BMI was negatively correlated with *Ruminococcus* and *Lactobacillus* (*Ruminococcus*: *r* = −0.147, *p* = 0.002, *Lactobacillus*: *r* = −0.153, *p* = 0.001). Grip/BMI was positively correlated with *Erysipelotrichaceae*, *Eggerthella*, and *Flavonifractor* in women (*Erysipelotrichaceae*: *r* = 0.128, *p* = 0.002; *Eggerthella*: *r* = 0.122, *p* = 0.003; *Flavonifractor*: *r* = 0.135, *p* = 0.001). In addition, grip/BMI was negatively correlated with *Faecalibacterium* (*r* = −0.129, *p* = 0.002).

## 4. Discussion

The aim of this study was to identify gut microbial genera associated with muscle strength in a large-scale survey considering health-related indicators. In the present study, the multiple regression analysis showed a positive association between *Eggerthella* and grip/BMI in both men and women. Three gut bacterial genera were found to be significantly associated with grip/BMI in both the multiple regression analysis and Spearman’s correlation analysis after a multiple comparison adjustment, setting the FDR threshold at 0.05. In particular, the increased relative counts of *Blautia* appeared to be associated with a higher grip/BMI in men. In addition, the increased relative counts of *Eggerthella* appeared to be associated with a higher grip/BMI in women.

A higher relative count of *Eggerthella* was associated with a higher grip/BMI in men and women in the multivariate analysis. Margiotta and Jackson reported that frail individuals had a higher relative count of *Eggerthella* compared to non-frail individuals in a study of the elderly [31,32,33]. Reports focusing on obese or frail individuals indicated that obese or overweight individuals had a lower relative count of *Eggerthella* compared to non-obese individuals [34]. Yun’s report supported the findings of our study. Reports on the association with *Eggerthella* varied depending on the study subjects, such as obese or frail individuals. In addition, previous study regarding the association between *Eggerthella* and muscle strength was not found. Further investigations are necessary.

A higher relative count of *Blautia* was also associated with a higher grip/BMI in men in the context of a multivariate analysis. Higher levels of *Blautia* were associated with a higher grip/BMI in women; nevertheless, the association was not significant. *Blautia* is a dominant intestinal bacterium that produces acetate. A study using myotube cells reported that acetate activates G-protein-coupled receptors and increases muscle-related proteins [35]. The authors previously revealed a positive association between *Blautia* and appendicular skeletal muscle mass/body weight in men [36]. Also, Ozato et al. demonstrated a negative association between *Blautia* and visceral fat area in both men and women in an obesity study [26]. The increase in *Blautia* may regulate the balance between skeletal muscle mass and body fat mass.

In addition, there have been some reports on the association between *Blautia* and exercise habits. In a study focusing on women, a positive correlation between *Blautia* and exercise intensity was reported [37]. Moreover, a report on childhood obesity, including both men and females, suggested that a 12-week exercise program combining strength training and aerobic exercise might lead to an increase in *Blautia* [38]. These findings indicate the importance of exercise habits that focus on intensity and duration. The exercise habits may increase *Blautia* and contribute to the improvement of muscle strength or the suppression of muscle weakness.

A higher relative count of *Faecalibacterium* was associated with grip/BMI in women. *Faecalibacterium* and short-chain fatty acids (butyrate) are positively related and regulate inflammatory effects [10]. The production of short-chain fatty acids by *Faecalibacterium* is mediated by interactions with other gut microbiota such as *Bifidobacterium* [39]. Bressa et al. reported that physically active women had higher levels of *Faecalibacterium* compared to sedentary women [40]. These findings contradict the results of this study. In this study, approximately half of the participants were taking medication, and the proportion of those with exercise habits was less than 30%. Grip/BMI values showed significantly lower values for individuals taking medication regardless of the presence of exercise habits. Therefore, the understanding of the results regarding the association between *Faecalibacterium* and muscle strength may have been complicated by the participants’ medication status.

The development of probiotic and prebiotic use for muscle strength is underway. Although there were some randomized trials on the probiotic supplementation on muscle strength [41], the three bacterial genera we found, *Eggerthella, Blautia, and Faecalibacterium,* were not used in the trials. Our findings suggest potential targets for the development of probiotics. While further exploration of causality is required, the findings from the current study offer evidence to inform future strategies for preventing muscle decline. *Faecalibacterium* and *Blautia* are associated with short-chain fatty acids [39,42]. Lustgarten speculates that higher fiber intake and more exercise may enhance the biosynthesis of short-chain fatty acids and the maintenance of a good gut microbiota, as well as muscle strength [43]. The enhanced biosynthesis of short-chain fatty acids by *Faecalibacterium* or *Blautia* may enhance exercise effectiveness and contribute to muscle weakness prevention.

This study has two strengths. First, it was a large-scale survey targeting a wide range of age groups in the local community. Second, by using grip/BMI, the influence of height and weight was minimized, enabling the examination of the association between the gut microbiota and muscle strength. However, there are several limitations to this study. First, this study was a cross-sectional study, and causality could not be confirmed. Second, materials in this study were obtained from a specific region, which may result in external validation problems. Third, despite the potential confounders we adjusted for in the multivariate analysis, unaccounted variables such as the specific types, volumes, and intensities of exercises could introduce bias. Therefore, further investigations utilizing tools that can objectively capture physical activity, such as accelerometers, are needed.

## 5. Conclusions

The study conducted a large-scale investigation across various age groups, targeting generally healthy individuals within the local community. *Eggerthella* was positively correlated with grip/BMI in men and women. *Blautia* was positively correlated with grip/BMI only in men. In addition, *Faecalibacterium* was negatively correlated with grip/BMI. To our knowledge, the relationship between grip/BMI and *Blautia* and *Eggerthella* has not been uncovered in previous studies. These gut microbial genera may help increase muscle strength or prevent muscle weakness. Whether causality exists between these gut microbial genera and muscle strength requires further investigation in future works.

## Figures and Tables

**Table 1:** Characteristics of the study participants.

	Men (*n* = 442)	Women (*n* = 588)
	Mean ± SD	Mean ± SD
Age (years)	50.5 ± 15.3	51.2 ± 15.9
**Body composition**		
Height (cm)	169.4 ± 6.5	156.8 ± 6.3
Weight (kg)	68.9 ± 11.5	54.8 ± 9.2
BMI (kg/m^2^)	24.0 ± 3.4	22.3 ± 3.5
**Muscle strength**		
Grip_Ave (kg)	41.5 ± 7.3	25.7 ± 4.2
Grip/BMI	1.8 ± 0.3	1.2 ± 0.2
**Nutrition**		
Protein (g/d)	74.8 ± 26	65.8 ± 24.3
Fat (g/d)	55.3 ± 20.2	50.9 ± 17.8
Carbohydrate (g/d)	281.0 ± 86.0	218.4 ± 68.7
Total dietary fiber (g/d)	11.0 ± 4.5	10.6 ± 4.2
Alcohol (g/d)	23.3 ± 26.8	5.7 ± 12.9
**Lifestyle**		
Habitual medicine use (no, %)	247 (55.9)	441 (50.5)
(yes, %)	195 (44.1)	291 (49.5)
Exercise (no, %)	320 (72.4)	429 (73.0)
(yes, %)	122 (27.6)	159 (27.0)
Smoking (no, %)	308 (69.7)	544 (92.5)
(yes, %)	134 (30.3)	44 (7.5)
Sleep time (min/day)	423.1 ± 69.2	409.4 ± 65.4

All values are expressed as mean ± standard deviation unless indicated otherwise. Abbreviations: SD: standard deviation, BMI = body mass index, BMI = body weight (kg)/height(m)^2^, g/d: grams per day.

**Table 2:** Gut microbiota composition.

Gut Microbiota		Men (*n* = 442)	Women (*n* = 588)	*p*-Value
		Mean ± SD	Mean ± SD	
**Read count**				
	Total lead	20,395.0 ± 5244.9	20,505.4 ± 5536.7	0.989
**Relative count (%)**				
	*Akkermansia*	0.52 ± 2.38	0.62 ± 1.80	<0.001
	*Alistipes*	1.42 ± 2.11	2.86 ± 3.41	<0.001
	*Clostridium IV*	1.85 ± 3.16	3.02 ± 4.16	<0.001
	*Collinsella*	5.62 ± 4.78	4.35 ± 4.74	<0.001
	*Dorea*	0.94 ± 0.83	0.76 ± 0.75	<0.001
	*Eggerthella*	0.14 ± 0.22	0.22 ± 0.30	<0.001
	*Faecalibacterium*	6.48 ± 5.16	8.14 ± 5.47	<0.001
	*Flavonifractor*	0.18 ± 0.20	0.25 ± 0.25	<0.001
	*Fusobacterium*	0.95 ± 3.26	0.28 ± 1.49	<0.001
	*Gemmiger*	2.11 ± 2.28	2.69 ± 2.58	<0.001
	*Intestinibacter*	0.16 ± 0.38	0.21 ± 0.34	<0.001
	*Lachnospiracea_incertae_sedis*	1.62 ± 1.04	1.96 ± 1.34	<0.001
	*Oscillibacter*	0.28 ± 0.36	0.45 ± 0.52	<0.001
	*Phascolarctobacterium*	0.71 ± 0.85	0.46 ± 0.67	<0.001
	*Prevotella*	7.51 ± 11.89	3.65 ± 8.61	<0.001
	*Ruminococcus*	2.45 ± 3.64	3.80 ± 4.77	<0.001
	*Sutterella*	1.36 ± 1.62	0.69 ± 1.04	<0.001
	*Butyricicoccus*	0.68 ± 0.51	0.62 ± 0.51	0.001
	*Dialister*	0.25 ± 0.51	0.27 ± 0.54	0.001
	*Slackia*	0.14 ± 0.38	0.08 ± 0.29	0.001
	*Megasphaera*	0.43 ± 1.25	0.28 ± 0.93	0.002
	*Odoribacter*	0.10 ± 0.22	0.14 ± 0.25	0.002
	*Megamonas*	1.29 ± 4.36	0.59 ± 2.21	0.004
	*Acidaminococcus*	0.19 ± 0.58	0.11 ± 0.39	0.010
	*Clostridium XlVa*	0.37 ± 0.33	0.43 ± 0.40	0.026
	*Mitsuokella*	0.17 ± 0.85	0.06 ± 0.45	0.051
	*Escherichia Shigella*	0.32 ± 1.71	0.40 ± 1.52	0.052
	*Bifidobacterium*	7.44 ± 8.17	8.10 ± 7.60	0.057
	*Erysipelotrichaceae_incertae_sedis*	0.43 ± 1.23	0.55 ± 1.66	0.061
	*Alloprevotella*	0.33 ± 1.34	0.13 ± 0.84	0.065
	*Klebsiella*	0.18 ± 1.16	0.04 ± 0.30	0.077
	*Paraprevotella*	0.25 ± 0.56	0.21 ± 0.47	0.089
	*Holdemanella*	0.87 ± 1.77	0.70 ± 1.68	0.094
	*Streptococcus*	1.75 ± 3.25	1.89 ± 3.56	0.100
	*Catenibacterium*	0.38 ± 1.37	0.29 ± 1.57	0.117
	*Parabacteroides*	0.96 ± 1.37	1.01 ± 1.17	0.138
	*Parasutterella*	0.41 ± 0.89	0.51 ± 0.94	0.264
	*Clostridium XlVb*	0.22 ± 0.31	0.19 ± 0.32	0.332
	*Ruminococcus2*	5.78 ± 5.84	5.24 ± 5.37	0.379
	*Bilophila*	0.14 ± 0.20	0.16 ± 0.21	0.389
	*Blautia*	7.41 ± 4.16	7.63 ± 4.19	0.392
	*Fusicatenibacter*	2.18 ± 2.11	2.38 ± 2.41	0.408
	*Bacteroides*	11.32 ± 7.26	11.75 ± 7.40	0.410
	*Enterococcus*	0.07 ± 0.41	0.13 ± 0.83	0.431
	*Clostridium XVIII*	0.77 ± 1.18	0.70 ± 0.96	0.440
	*Veillonella*	0.64 ± 1.74	0.67 ± 1.43	0.479
	*Coprococcus*	0.54 ± 0.69	0.57 ± 0.80	0.482
	*Turicibacter*	0.30 ± 1.42	0.18 ± 0.43	0.527
	*Clostridium sensu stricto*	0.23 ± 0.70	0.17 ± 0.46	0.550
	*Barnesiella*	0.09 ± 0.25	0.13 ± 0.30	0.651
	*Lactobacillus*	0.52 ± 2.80	0.19 ± 0.83	0.653
	*Romboutsia*	0.29 ± 0.57	0.29 ± 0.63	0.666
	*Anaerostipes*	5.51 ± 5.91	5.04 ± 5.12	0.671
	*Roseburia*	3.97 ± 3.86	3.94 ± 3.74	0.897

Mean ± standard deviations are presented for continuous variables. *p*-values are presented for the differences between men and women. *p* < 0.05, Mann–Whitney U-test.

**Table 3:** Association between gut microbiota and grip/BMI.

Gut Microbiota	Men (*n* = 442)		Women (*n* = 588)	
	*β* (95%CI)	*p*-Value	*β* (95%CI)	*p*-Value
*Acidaminococcus*	−0.12 (−0.17, −0.07)	<0.001	−0.01 (−0.06, 0.03)	0.549
*Allisonella*	−0.83 (−1.36, −0.29)	0.003	−0.35 (−0.89, 0.19)	0.201
*Blautia*	0.01 (0.00, 0.02)	0.021	0.00 (−0.00, 0.01)	0.666
*Clostridium XVIII*	0.03 (0.00, 0.05)	0.038	−0.01 (−0.03, 0.01)	0.236
*Collinsella*	−0.01 (−0.02, −0.00)	0.005	0.00 (−0.00, 0.00)	0.926
*Dialister*	−0.07 (−0.13, −0.01)	0.023	−0.02 (−0.05, 0.01)	0.189
*Eggerthella*	0.18 (0.04, 0.31)	0.009	0.07 (0.00, 0.12)	0.028
*Erysipelotrichaceae_incertae_sedis*	0.03 (0.00, 0.05)	0.027	−0.01 (−0.00, 0.01)	0.611
*Escherichia Shigella*	0.02 (0.00, 0.04)	0.041	−0.00 (−0.02, 0.00)	0.129
*Faecalibacterium*	0.00 (−0.00, 0.01)	0.459	−0.01 (−0.01, −0.00)	0.003
*Parasutterella*	−0.04 (−0.07, −0.00)	0.036	−0.03 (−0.06, 0.01)	0.168
*Ruminococcus*	−0.01 (−0.02, 0.00)	0.157	0.00 (0.00, 0.01)	0.047

This multivariate analysis was adjusted for age, medicine, smoking, exercise, sleep time, protein, fat, carbohydrate, total dietary fiber, and alcohol.

## Data Availability

The data presented in this study are available upon request from the corresponding author. The data are not publicly available due to the test sample patent and the participants’ privacy and confidentiality.
